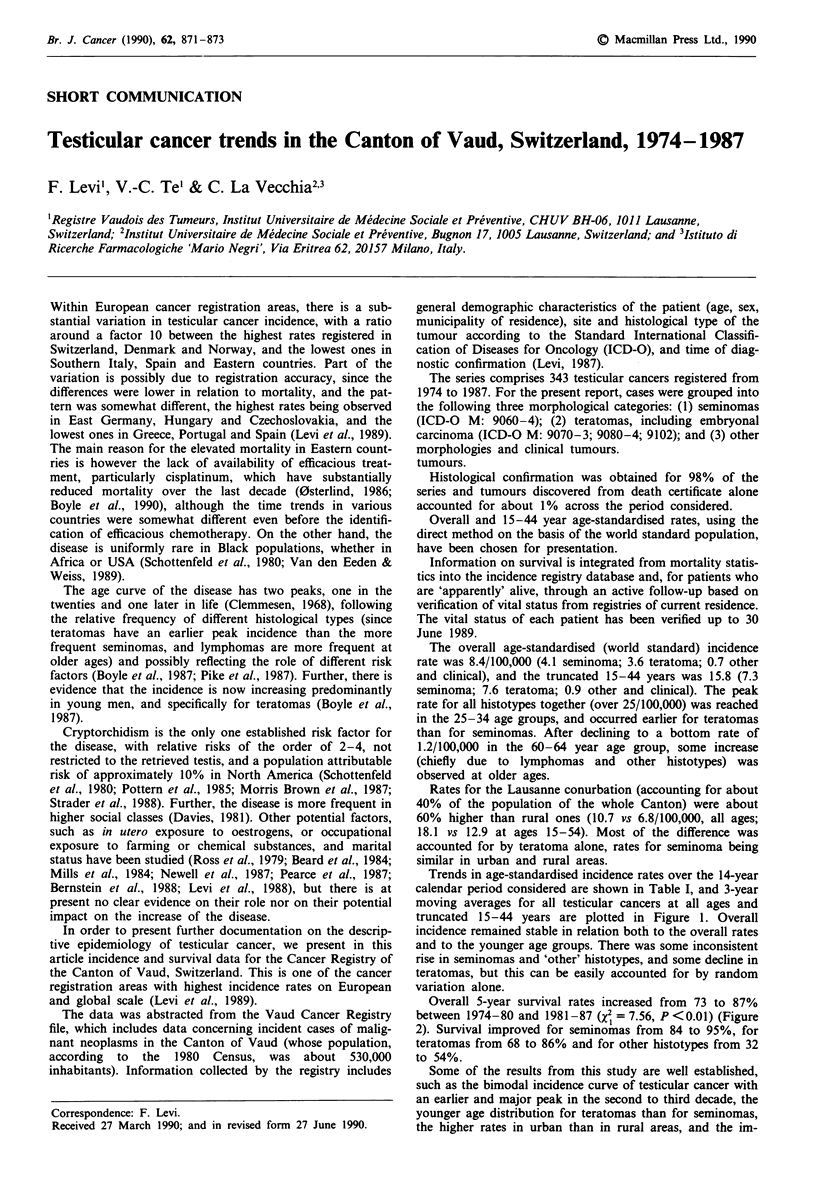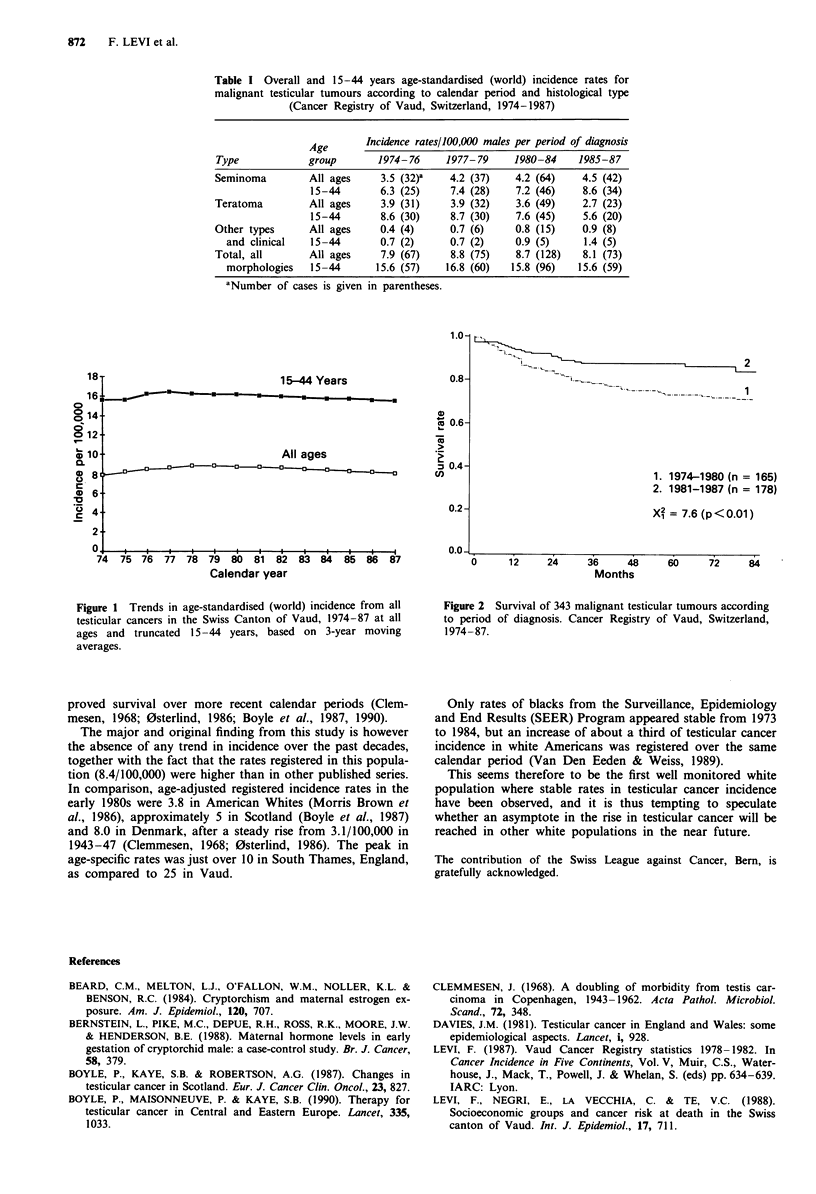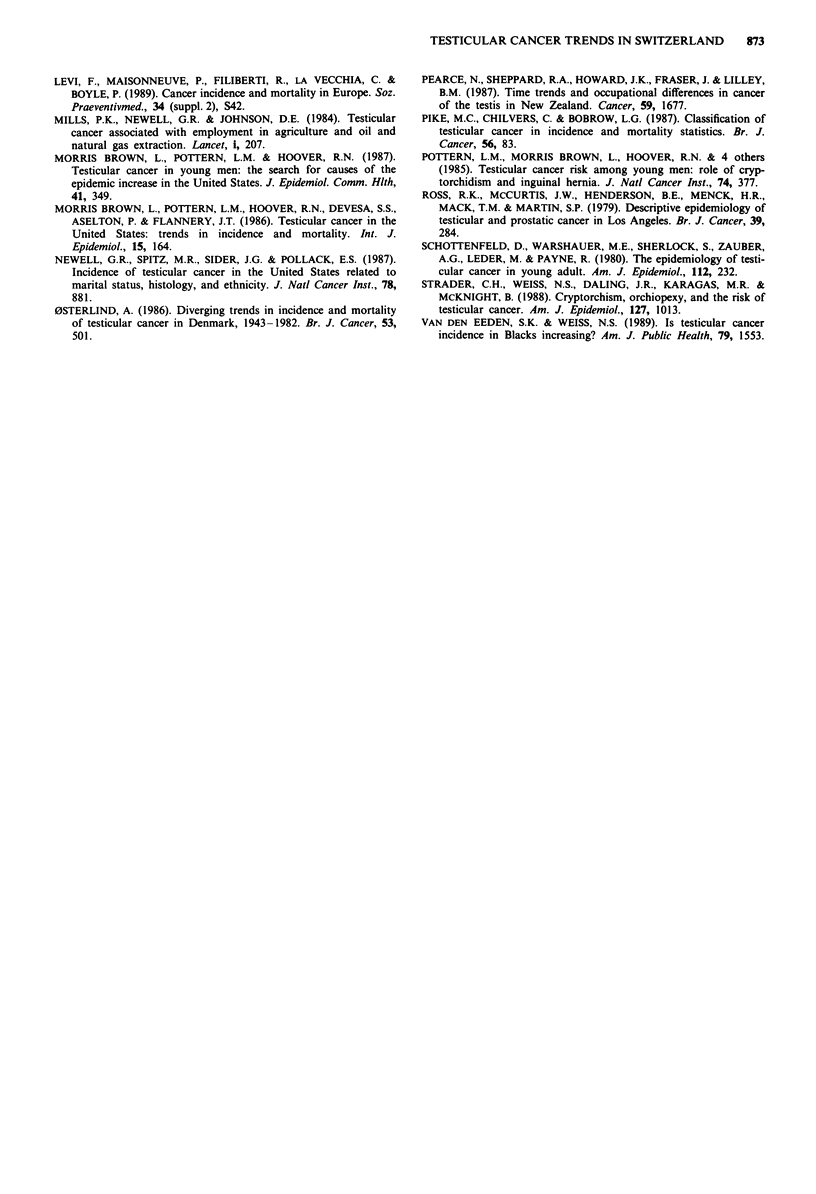# Testicular cancer trends in the Canton of Vaud, Switzerland, 1974-1987.

**DOI:** 10.1038/bjc.1990.398

**Published:** 1990-11

**Authors:** F. Levi, V. C. Te, C. La Vecchia

**Affiliations:** Registre Vaudois des Tumeurs, Institut Universitaire de Médecine Sociale et Préventive, CHUV, Lausanne, Switzerland.


Br. J. Cancer (1990), 62, 871-873 © Macmillan Press Ltd., 1990

SHORT COMMUNICATION

### Testicular cancer trends in the Canton of Vaud, Switzerland, 1974-1987

F. Levi[1], V.-C. Te[1] & C. La Vecchia[2,3]

[1]*Registre Vaudois des Tumeurs, Institut Universitaire de Médecine Sociale et Préventive, CHUV BH-06, 1011 Lausanne, Switzerland;* [2]*Institut Universitaire de Médecine Sociale et Préventive, Bugnon 17, 1005 Lausanne, Switzerland; and* [3]*Istituto di Ricerche Farmacologiche 'Mario Negri', Via Eritrea 62, 20157 Milano, Italy.*

Within European cancer registration areas, there is a substantial variation in testicular cancer incidence, with a ratio around a factor 10 between the highest rates registered in Switzerland, Denmark and Norway, and the lowest ones in Southern Italy, Spain and Eastern countries. Part of the variation is possibly due to registration accuracy, since the differences were lower in relation to mortality, and the pattern was somewhat different, the highest rates being observed in East Germany, Hungary and Czechoslovakia, and the lowest ones in Greece, Portugal and Spain (Levi *et al.*, 1989). The main reason for the elevated mortality in Eastern countries is however the lack of availability of efficacious treatment, particularly cisplatinum, which have substantially reduced mortality over the last decade (Østerlind, 1986; Boyle *et al.*, 1990), although the time trends in various countries were somewhat different even before the identification of efficacious chemotherapy. On the other hand, the disease is uniformly rare in Black populations, whether in Africa or USA (Schottenfeld *et al.*, 1980; Van den Eeden & Weiss, 1989).

The age curve of the disease has two peaks, one in the twenties and one later in life (Clemmesen, 1968), following the relative frequency of different histological types (since teratomas have an earlier peak incidence than the more frequent seminomas, and lymphomas are more frequent at older ages) and possibly reflecting the role of different risk factors (Boyle *et al.*, 1987; Pike *et al.*, 1987). Further, there is evidence that the incidence is now increasing predominantly in young men, and specifically for teratomas (Boyle *et al.*, 1987).

Cryptorchidism is the only one established risk factor for the disease, with relative risks of the order of 2-4, not restricted to the retrieved testis, and a population attributable risk of approximately 10% in North America (Schottenfeld *et al.*, 1980; Pottern *et al.*, 1985; Morris Brown *et al.*, 1987; Strader *et al.*, 1988). Further, the disease is more frequent in higher social classes (Davies, 1981). Other potential factors, such as *in utero* exposure to oestrogens, or occupational exposure to farming or chemical substances, and marital status have been studied (Ross *et al.*, 1979; Beard *et al.*, 1984; Mills *et al.*, 1984; Newell *et al.*, 1987; Pearce *et al.*, 1987; Bernstein *et al.*, 1988; Levi *et al.*, 1988), but there is at present no clear evidence on their role nor on their potential impact on the increase of the disease.

In order to present further documentation on the descriptive epidemiology of testicular cancer, we present in this article incidence and survival data for the Cancer Registry of the Canton of Vaud, Switzerland. This is one of the cancer registration areas with highest incidence rates on European and global scale (Levi *et al.*, 1989).

The data was abstracted from the Vaud Cancer Registry file, which includes data concerning incident cases of malignant neoplasms in the Canton of Vaud (whose population, according to the 1980 Census, was about 530,000 inhabitants). Information collected by the registry includes general demographic characteristics of the patient (age, sex, municipality of residence), site and histological type of the tumour according to the Standard International Classification of Diseases for Oncology (ICD-O), and time of diagnostic confirmation (Levi, 1987).

The series comprises 343 testicular cancers registered from 1974 to 1987. For the present report, cases were grouped into the following three morphological categories: (1) seminomas (ICD-O M: 9060-4); (2) teratomas, including embryonal carcinoma (ICD-O M: 9070-3; 9080-4; 9102); and (3) other morphologies and clinical tumours.
tumours.

Histological confirmation was obtained for 98% of the series and tumours discovered from death certificate alone accounted for about 1% across the period considered.

Overall and 15-44 year age-standardised rates, using the direct method on the basis of the world standard population, have been chosen for presentation.

Information on survival is integrated from mortality statistics into the incidence registry database and, for patients who are 'apparently' alive, through an active follow-up based on verification of vital status from registries of current residence. The vital status of each patient has been verified up to 30 June 1989.

The overall age-standardised (world standard) incidence rate was 8.4/100,000 (4.1 seminoma; 3.6 teratoma; 0.7 other and clinical), and the truncated 15-44 years was 15.8 (7.3 seminoma; 7.6 teratoma; 0.9 other and clinical). The peak rate for all histotypes together (over 25/100,000) was reached in the 25-34 age groups, and occurred earlier for teratomas than for seminomas. After declining to a bottom rate of 1.2/100,000 in the 60-64 year age group, some increase (chiefly due to lymphomas and other histotypes) was observed at older ages.

Rates for the Lausanne conurbation (accounting for about 40% of the population of the whole Canton) were about 60% higher than rural ones (10.7 *vs* 6.8/100,000, all ages; 18.1 *vs* 12.9 at ages 15-54). Most of the difference was accounted for by teratoma alone, rates for seminoma being similar in urban and rural areas.

Trends in age-standardised incidence rates over the 14-year calendar period considered are shown in Table I, and 3-year moving averages for all testicular cancers at all ages and truncated 15-44 years are plotted in Figure 1. Overall incidence remained stable in relation both to the overall rates and to the younger age groups. There was some inconsistent rise in seminomas and 'other' histotypes, and some decline in teratomas, but this can be easily accounted for by random variation alone.

Overall 5-year survival rates increased from 73 to 87% between 1974-80 and 1981-87 ($\chi^2_1 = 7.56$, $P < 0.01$) (Figure 2). Survival improved for seminomas from 84 to 95%, for teratomas from 68 to 86% and for other histotypes from 32 to 54%.

Some of the results from this study are well established, such as the bimodal incidence curve of testicular cancer with an earlier and major peak in the second to third decade, the younger age distribution for teratomas than for seminomas, the higher rates in urban than in rural areas, and the im-

Correspondence: F. Levi.
Received 27 March 1990; and in revised form 27 June 1990.

**Table I** Overall and 15–44 years age-standardised (world) incidence rates for malignant testicular tumours according to calendar period and histological type (Cancer Registry of Vaud, Switzerland, 1974–1987)

| Type | Age group | *Incidence rates/100,000 males per period of diagnosis* 1974–76 | 1977–79 | 1980–84 | 1985–87 |
|---|---|---|---|---|---|
| Seminoma | All ages | 3.5 (32)[a] | 4.2 (37) | 4.2 (64) | 4.5 (42) |
| | 15–44 | 6.3 (25) | 7.4 (28) | 7.2 (46) | 8.6 (34) |
| Teratoma | All ages | 3.9 (31) | 3.9 (32) | 3.6 (49) | 2.7 (23) |
| | 15–44 | 8.6 (30) | 8.7 (30) | 7.6 (45) | 5.6 (20) |
| Other types | All ages | 0.4 (4) | 0.7 (6) | 0.8 (15) | 0.9 (8) |
| and clinical | 15–44 | 0.7 (2) | 0.7 (2) | 0.9 (5) | 1.4 (5) |
| Total, all | All ages | 7.9 (67) | 8.8 (75) | 8.7 (128) | 8.1 (73) |
| morphologies | 15–44 | 15.6 (57) | 16.8 (60) | 15.8 (96) | 15.6 (59) |

[a]Number of cases is given in parentheses.

**Figure 1** Trends in age-standardised (world) incidence from all testicular cancers in the Swiss Canton of Vaud, 1974–87 at all ages and truncated 15–44 years, based on 3-year moving averages.

**Figure 2** Survival of 343 malignant testicular tumours according to period of diagnosis. Cancer Registry of Vaud, Switzerland, 1974–87.

proved survival over more recent calendar periods (Clemmesen, 1968; Østerlind, 1986; Boyle *et al.*, 1987, 1990).

The major and original finding from this study is however the absence of any trend in incidence over the past decades, together with the fact that the rates registered in this population (8.4/100,000) were higher than in other published series. In comparison, age-adjusted registered incidence rates in the early 1980s were 3.8 in American Whites (Morris Brown *et al.*, 1986), approximately 5 in Scotland (Boyle *et al.*, 1987) and 8.0 in Denmark, after a steady rise from 3.1/100,000 in 1943–47 (Clemmesen, 1968; Østerlind, 1986). The peak in age-specific rates was just over 10 in South Thames, England, as compared to 25 in Vaud.

Only rates of blacks from the Surveillance, Epidemiology and End Results (SEER) Program appeared stable from 1973 to 1984, but an increase of about a third of testicular cancer incidence in white Americans was registered over the same calendar period (Van Den Eeden & Weiss, 1989).

This seems therefore to be the first well monitored white population where stable rates in testicular cancer incidence have been observed, and it is thus tempting to speculate whether an asymptote in the rise in testicular cancer will be reached in other white populations in the near future.

The contribution of the Swiss League against Cancer, Bern, is gratefully acknowledged.

### References

BEARD, C.M., MELTON, L.J., O'FALLON, W.M., NOLLER, K.L. & BENSON, R.C. (1984). Cryptorchism and maternal estrogen exposure. *Am. J. Epidemiol.*, **120,** 707.

BERNSTEIN, L., PIKE, M.C., DEPUE, R.H., ROSS, R.K., MOORE, J.W. & HENDERSON, B.E. (1988). Maternal hormone levels in early gestation of cryptorchid male: a case-control study. *Br. J. Cancer*, **58,** 379.

BOYLE, P., KAYE, S.B. & ROBERTSON, A.G. (1987). Changes in testicular cancer in Scotland. *Eur. J. Cancer Clin. Oncol.*, **23,** 827.

BOYLE, P., MAISONNEUVE, P. & KAYE, S.B. (1990). Therapy for testicular cancer in Central and Eastern Europe. *Lancet*, **335,** 1033.

CLEMMESEN, J. (1968). A doubling of morbidity from testis carcinoma in Copenhagen, 1943–1962. *Acta Pathol. Microbiol. Scand.*, **72,** 348.

DAVIES, J.M. (1981). Testicular cancer in England and Wales: some epidemiological aspects. *Lancet*, **i,** 928.

LEVI, F. (1987). Vaud Cancer Registry statistics 1978–1982. In *Cancer Incidence in Five Continents*, Vol. V, Muir, C.S., Waterhouse, J., Mack, T., Powell, J. & Whelan, S. (eds) pp. 634–639. IARC: Lyon.

LEVI, F., NEGRI, E., LA VECCHIA, C. & TE, V.C. (1988). Socioeconomic groups and cancer risk at death in the Swiss canton of Vaud. *Int. J. Epidemiol.*, **17,** 711.

LEVI, F., MAISONNEUVE, P., FILIBERTI, R., LA VECCHIA, C. & BOYLE, P. (1989). Cancer incidence and mortality in Europe. *Soz. Praeventivmed.*, **34** (suppl. 2), S42.

MILLS, P.K., NEWELL, G.R. & JOHNSON, D.E. (1984). Testicular cancer associated with employment in agriculture and oil and natural gas extraction. *Lancet*, **i**, 207.

MORRIS BROWN, L., POTTERN, L.M. & HOOVER, R.N. (1987). Testicular cancer in young men: the search for causes of the epidemic increase in the United States. *J. Epidemiol. Comm. Hlth*, **41**, 349.

MORRIS BROWN, L., POTTERN, L.M., HOOVER, R.N., DEVESA, S.S., ASELTON, P. & FLANNERY, J.T. (1986). Testicular cancer in the United States: trends in incidence and mortality. *Int. J. Epidemiol.*, **15**, 164.

NEWELL, G.R., SPITZ, M.R., SIDER, J.G. & POLLACK, E.S. (1987). Incidence of testicular cancer in the United States related to marital status, histology, and ethnicity. *J. Natl Cancer Inst.*, **78**, 881.

ØSTERLIND, A. (1986). Diverging trends in incidence and mortality of testicular cancer in Denmark, 1943–1982. *Br. J. Cancer*, **53**, 501.

PEARCE, N., SHEPPARD, R.A., HOWARD, J.K., FRASER, J. & LILLEY, B.M. (1987). Time trends and occupational differences in cancer of the testis in New Zealand. *Cancer*, **59**, 1677.

PIKE, M.C., CHILVERS, C. & BOBROW, L.G. (1987). Classification of testicular cancer in incidence and mortality statistics. *Br. J. Cancer*, **56**, 83.

POTTERN, L.M., MORRIS BROWN, L., HOOVER, R.N. & 4 others (1985). Testicular cancer risk among young men: role of cryptorchidism and inguinal hernia. *J. Natl Cancer Inst.*, **74**, 377.

ROSS, R.K., MCCURTIS, J.W., HENDERSON, B.E., MENCK, H.R., MACK, T.M. & MARTIN, S.P. (1979). Descriptive epidemiology of testicular and prostatic cancer in Los Angeles. *Br. J. Cancer*, **39**, 284.

SCHOTTENFELD, D., WARSHAUER, M.E., SHERLOCK, S., ZAUBER, A.G., LEDER, M. & PAYNE, R. (1980). The epidemiology of testicular cancer in young adult. *Am. J. Epidemiol.*, **112**, 232.

STRADER, C.H., WEISS, N.S., DALING, J.R., KARAGAS, M.R. & MCKNIGHT, B. (1988). Cryptorchism, orchiopexy, and the risk of testicular cancer. *Am. J. Epidemiol.*, **127**, 1013.

VAN DEN EEDEN, S.K. & WEISS, N.S. (1989). Is testicular cancer incidence in Blacks increasing? *Am. J. Public Health*, **79**, 1553.